# Fetal sex-specific differences in gestational age at delivery in pre-eclampsia: a meta-analysis

**DOI:** 10.1093/ije/dyw178

**Published:** 2016-09-06

**Authors:** Sarah Schalekamp-Timmermans, Lidia R Arends, Elin Alsaker, Lucy Chappell, Stefan Hansson, Nina K Harsem, Maya Jälmby, Arundhathi Jeyabalan, Hannele Laivuori, Debbie A Lawlor, Corrie Macdonald-Wallis, Per Magnus, Jenny Myers, Jørn Olsen, Lucilla Poston, Christopher W Redman, Anne C Staff, Pia Villa, James M Roberts, Eric A Steegers, On behalf of the Global Pregnancy Collaboration:

**Affiliations:** 1 Erasmus Medical Centre, Department of Obstetrics and Gynecology, Rotterdam, The Netherlands,; 2 Institute of Psychology, and Department of Pedagogical Sciences, Erasmus University Rotterdam, Rotterdam, The Netherlands, and Erasmus Medical Centre, Department of Biostatistics, Rotterdam, The Netherlands,; 3 Norwegian Institute of Public Health, Oslo, Norway,; 4 Women’s Health Academic Centre, King’s College London and King’s Health Partners, London, UK,; 5 Lund University, Department of Clinical Sciences, Obstetrics and Gynecology, Lund, Sweden, and Skåne University Hospital, Perinatal Unit, Malmo, Sweden,; 6 Oslo University Hospital, Department of Obstetrics, Oslo, Norway,; 7 Lund University, Department of Clinical Sciences, Obstetrics and Gynecology, Lund, Sweden, and Skåne University Hospital, Department of Obstetrics and Gynecology, Malmo, Sweden,; 8 University of Pittsburgh School of Medicine, Division of Maternal-Fetal Medicine, Department of Obstetrics, Gynecology, and Reproductive Sciences, Pittsburgh, PA, USA,; 9 Medical and Clinical Genetics and Obstetrics and Gynecology, and Institute for Molecular Medicine Finland, University of Helsinki, Helsinki, Finland,; 10 MRC Integrative Epidemiology Unit at the University of Bristol, School of Social and Community Medicine, Bristol, UK,; 11 Maternal & Fetal Health Research Centre, Manchester Academic Health Science Centre, University of Manchester, Manchester, UK,; 12 Aarhus University, Institute of Clinical Epidemiology, Aarhus, Denmark, and UCLA Los Angeles, Los Angeles, CA, USA,; 13 Nuffield Department of Obstetrics and Gynecology, John Radcliffe Hospital, Oxford, UK,; 14 Oslo University Hospital, Department of Obstetrics and Department of Gynecology, University of Oslo, Oslo, Norway,; 15 Obstetrics and Gynecology, and Clinical Graduate School in Pediatrics and Obstetrics/Gynecology, University of Helsinki, Helsinki, Finland,; 16 Department of Obstetrics, Gynecology and Reproductive Sciences, Epidemiology and Clinical and Translational Research University of Pittsburgh, Pittsburgh, PA, USA

**Keywords:** Sexual dimorphism, pre-eclampsia, placenta, sex ratio, ALSPAC

## Abstract

**Background**: Pre-eclampsia (PE) is a major pregnancy disorder complicating up to 8% of pregnancies. Increasing evidence indicates a sex-specific interplay between the mother, placenta and fetus. This may lead to different adaptive mechanisms during pregnancy.

**Methods:** We performed an individual participant data meta-analysis to determine associations of fetal sex and PE, with specific focus on gestational age at delivery in PE. This was done on 219 575 independent live-born singleton pregnancies, with a gestational age at birth between 22.0 and 43.0 weeks of gestation, from 11 studies participating in a worldwide consortium of international research groups focusing on pregnancy.

**Results:** Of the women, 9033 (4.1%) experienced PE in their pregnancy and 48.8% of the fetuses were female versus 51.2% male. No differences in the female/male distribution were observed with respect to term PE (delivered ≥ 37 weeks). Preterm PE (delivered < 37 weeks) was slightly more prevalent among pregnancies with a female fetus than in pregnancies with a male fetus [odds ratio (OR) 1.11, 95% confidence interval (CI) 1.02–1.21]. Very preterm PE (delivered < 34 weeks) was even more prevalent among pregnancies with a female fetus as compared with pregnancies with a male fetus (OR 1.36, 95% CI 1.17–1.59).

**Conclusions:** Sexual dimorphic differences in the occurrence of PE exist, with preterm PE being more prevalent among pregnancies with a female fetus as compared with pregnancies with a male fetus and with no differences with respect to term PE.

## Introduction

There are known large sex differences in disease incidence, presentation, diagnosis and outcome to treatment.^1^ During past years attention has focused on the female/male distribution during pregnancy and its interaction with maternal health. Apparently, maternal physiological functions are influenced in a fetal sex-specific manner during pregnancy.^2^ Pre-eclampsia (PE) is a major pregnancy disorder complicating up to 8% of pregnancies in some countries. PE is an important contributor to maternal and perinatal morbidity and mortality worldwide.^3^ Pre-eclamptic women as well as their children have an increased risk to develop cardiovascular disease and stroke later in life.^4^ A previous study indicated that fetal sex influenced gestational age at delivery in a Norwegian population from up to 50 years ago, with female fetuses predominating in pre-eclamptic pregnancies ending before 37 weeks.^5^ Gestational age has been suggested as an indicator of subsets of PE with a different pathophysiology and with different acute and long-range outcomes for both mother and baby. Therefore, in this study we sought to confirm and extend these earlier findings to very preterm pregnancies in a more diverse and contemporary pregnancy population. To assess sex-specific differences in gestational age at delivery in pre-eclamptic pregnancies, we conducted a meta-analysis of individual data from 219 575 pregnant women participating in 11 studies from several European, Oceanian and US centres.

## Material and Methods

### Inclusion criteria and participating cohorts

In 2011, the Global Pregnancy Collaboration (CoLab) was established to facilitate data and sample sharing between research groups studying PE and other pregnancy disorders [pre-empt.cfri.ca/Collaboration/global-pregnancy-Collaboration]. CoLab is a consortium of international research groups with data and biological samples from women before, during and in some cases long after pregnancy. Information on clinical data and samples is offered in a membership-wide shared database and available to CoLab members and to investigators sponsored by CoLab members.^6^ In 2012, we invited principal investigators of international research groups active in CoLab to participate in the current study. Studies participated if they included pregnant women with available information on the occurrence of PE. Information on gestational age at birth and fetal sex also had to be available. Only live-born singleton pregnancies with a gestational age at birth between 22.0 and 43.0 weeks of gestation were included. Both nulliparous and multiparous women could participate. Eleven studies agreed to participate, comprising 219 575 independent singleton pregnancies that met the inclusion criteria.^7–16^ The studies varied in sample size as well as study design, including both low- and high-risk pregnancies. Study-specific information with references to detailed information about each individual study is shown in Table 1

**Table 1.** Characteristics of the participating studies

Study	Country	Design	Setting	No. (= 219575)	Year inclusion	PE, *n* (% within study)
Avon Longitudinal Study of Parents and Children^7^	UK	Prospective cohort study	Population based	13444	1991–92	317 (2.4)
Danish National Birth Cohort (DNBC)^8^	Denmark	Prospective cohort study	Population based	83532	1996–2002	2040 (2.4)
Finnish Genetics of Pre-eclampsia Consortium^9^	Finland	Case-control study	Hospital based	1930	2008–12	1049 (54.4)
Generation R Study (GenR)^10^	The Netherlands	Prospective cohort study	Population based	8363	2002–06	198 (2.4)
Lund Database (Lund)	Sweden	Prospective cohort study	Hospital based	545	1999-–014	239 (43.8)
The Norwegian Mother and Child Cohort (MoBa)^11^	Norway	Prospective cohort study	Population based	98436	1999–2009	3721 (3.8)
Oslo Pregnancy Biobank (OPB)^12^	Norway	Case-control study	Hospital based	472	2001–13	182 (38.6)
Pregnancy Exposures and Preeclampsia Prevention Study (PEPP)^13^	USA	Prospective cohort study	Population based	4274	1999–2007	597 (14.0)
Prediction and Prevention of Pre-eclampsia Project (PREDO)^14^	Finland	Prospective cohort study	Hospital based	1032	2005–09	92 (8.9)
The Screening for Pregnancy Endpoints (SCOPE)^15^	New Zealand, Australia, UK and Ireland	Prospective cohort study	Hospital based	5573	2004–11	275 (4.9)
Vitamin C and Vitamin E in Pregnant Women at Risk for Pre-Eclampsia trial (VIP)^16^	UK	Randomized clinical trial	Hospital based	1974	2003–05	323 (16.4)

Studies are listed in alphabetical order.

*No.*, number of participants with a live-born singleton pregnancy between 22–43 weeks of gestation and complete information on the occurrence of pre-eclampsia (PE) and fetal sex.

. All studies were approved by the national, regional and local relevant research review boards. Regarding the ALSPAC study, ethical approval for the study was obtained from the ALSPAC Ethics and Law Committee and the local research ethics committees. All participants provided written informed consent for use of their data. Anonymized data sets were stored on a single central secured data server with access for the main analysts only. MOOSE guidelines for reporting a meta-analysis were followed.

### Pre-eclampsia

Information on PE per study was obtained per participating centre by using measurements, medical registries, hospital records and/or specific questionnaires. Gestational hypertension was defined as a blood pressure > 140 mmHg systolic or > 90 mmHg diastolic in a woman who was normotensive before 20 weeks’ gestation without concurrent new-onset proteinuria. In all studies participating in CoLab and in this study, PE is defined according to former International Society for the Study of Hypertension in Pregnancy criteria (*de novo* gestational hypertension with concurrent new-onset proteinuria [≥ 0.3 g protein in a 24-h specimen, correlating with ≥ 30 mg/dl (≥ 1 + reading on dipstick) in a random urine determination with no evidence of urinary tract infection].^17^ Superimposed PE was defined as chronic hypertension diagnosed before pregnancy or in the first 20 weeks of pregnancy, complicated by *de novo* proteinuria occurring after gestational week 20, in the absence of renal disease and urinary tract infection. As PE is a syndrome that does not necessarily present as *de novo* hypertension and proteinuria the same day, and as routine antenatal follow-up schedules differ between countries and pregnancies, the time of PE diagnosis is difficult to define precisely. Instead, gestational age at delivery was used as a proxy for the onset of disease. Women with a very early onset of PE (before gestational week 34) often present with combined intrauterine growth restriction (IUGR) or rapidly increasing maternal symptoms and rarely remain undelivered for many days or weeks. Women with term PE (from gestational week 37 + 0) are likely to be induced (provided vaginal delivery is feasible and clinically justified) and delivered shortly after diagnosis, complying with current international clinical PE guidelines. As gestational age at delivery was reliably registered in the centres that were included in this analysis, this was used as a proxy to distinguish between term, preterm and very preterm PE (i.e. delivery ≥ 37 + 0 weeks of gestation, < 37 weeks of gestation and < 34 weeks of gestation). This distinction between early and very early versus term ‘onset’ of PE is a commonly used categorization in PE studies.

### Covariates

Information about maternal characteristics (maternal age, parity, body mass index and the presence of chronic hypertension) and birth characteristics (gestational age at birth, offspring birthweight and fetal sex) in each study was obtained per participating centre by using measurements, medical registries, hospital records and/or specific questionnaires.

### Statistical analyses

Individual datasets were integrated into one central database. For the cleaning of the central database the following criteria were used: values had to be within three standard deviations at either side of the mean and/or values had to be clinically reasonable. Random-effects models as proposed by DerSimonian and Laird were used to take the potential between-study variation next to the within-study variation into account.^18^^,^^19^ In this model, the inverse of standard errors from the individual studies combined with the between-study variation were used as weights. Heterogeneity was assessed by the I^2^ index. The I^2^ index describes the proportion of total variation in the effect sizes that is due to heterogeneity between studies. To determine the influence of any particular cohort on overall results, we repeated each meta-analysis, leaving out one cohort at a time (leave-one-out methodology). The overall effects are presented as forest plots with the pooled odds ratios from the random-effects models with 95% confidence intervals (CI). Statistical analyses were performed with SAS 9.2 software (SAS Institute, Cary, NC) and Comprehensive Meta-Analysis 2.0 (Biostat, Englewood, USA).

## Results

### Subject characteristics

Study-specific information about maternal and birth characteristics is shown in Table 2

**Table 2.** Maternal and birth characteristics

	Total cohort	Alspac	DNBC	FINNPEC	GenR	Lund
	*N = *219575	*n = *13444	*n = *83532	*n = *1930	*n = *8363	*n* = 545
Maternal age, years (mean, SD)	29.8 (4.7)	28.0 (5.0)	29.8 (4.4)	29.9 (5.4)	29.7 (5.3)	30.0 (5.0)
Parity, % 0	50.4	45	50.6	66.1	55.0	68.5
BMI, kg/m^2^	23.0	21.6	22.6	23.6	23.9	NA
(median, 90% range)	(18.7–32.9)	(17.6–30.7)	(18.6–31.9)	(19.1–34.4)	(19.4–33.7)	NA
Chronic hypertension, % yes	1.3	3.8	0.2	10.6	1.9	0.9
Gestational age birth, weeks	40.0	40.0	40.0	39.0	40.1	38.7
(median, 90% range)	(36.0–42.0)	(36.0–42.0)	(37.0–42.0)	(31.0-–42.0)	(36.9–42.0)	(29.2–41.7)
Birthweight, grams (mean, SD)	3547.5 (585.0)	3408.7 (551.5)	3574.3 (571.9)	3096.3 (861.6)	3411.9 (561.5)	3156.1 (866.2)
Fetal sex, % female	48.8	48.4	48.8	50.8	49.5	50.3

	MoBa	OPB	PEPP	PREDO	SCOPE	VIP
	*n* = 98436	*n* = 472	*n* = 4274	*n* = 1032	*n* = 5573	*n* = 1974
Maternal age, years (mean, SD)	30.2 (4.6)	31.7 (4.9)	26.3 (6.3)	32.3 (5.8)	28.7 (5.5)	30.8 (5.9)
Parity, % 0	46.7	53.8	68.1	31.8	100	49.9
BMI, kg/m^2^	23.1	23.9	24.1	25.5	24.2	31.2
(median, 90% range)	(18.9–32.5)	(19.1–35.9)	(18.1–39.2)	(19.1–39.5)	(19.5–34.7)	(21.1–43.4)
Chronic hypertension, % yes	0.5	0.2	2.5	18.4	2.7	41.0
Gestational age birth, weeks	40.0	38.4	39.0	39.9	40.1	39.0
(median, 90% range)	(37.0–42.0)	(28.6–40.3)	(33.0–41.0)	(36.4–41.9)	(36.6–41.7)	(33.1–41.9)
Birthweight, grams (mean, SD)	3600.3 (560.5)	3129.5 (1015.8)	3141.0 (728.3)	3510.7 (597.6)	3415.6 (555.4)	3217.7 (761.2)
Fetal sex, % female	48.7	50	49.2	46.6	49.2	49.1


NA, not available.

. The overall distribution of female and male fetuses was 48.8% versus 51.2%. The overall prevalences of gestational hypertension and PE were 2.9% and 4.1% (*n* = 6150 and *n* = 9033), respectively. Of the pre-eclamptic women, 6.4% had superimposed PE (*n* = 575). Of the remaining 8458 *de novo* pre-eclamptic women, 15.4% were diagnosed with very preterm PE (<34 weeks of gestation, *n* = 1306).

### Pre-eclampsia and fetal sex

**Figure 1.**
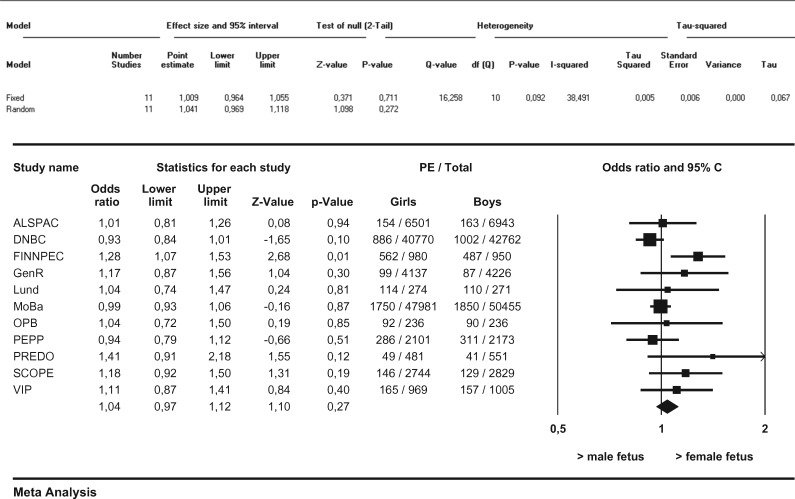
Associations between fetal sex and *overall* PE between female and male pregnancies Results from random-effects models. Data reflect Odds ratios (95% Confidence Interval) in which female preeclampsia (PE) is compared to male PE.

**Figure 2.**
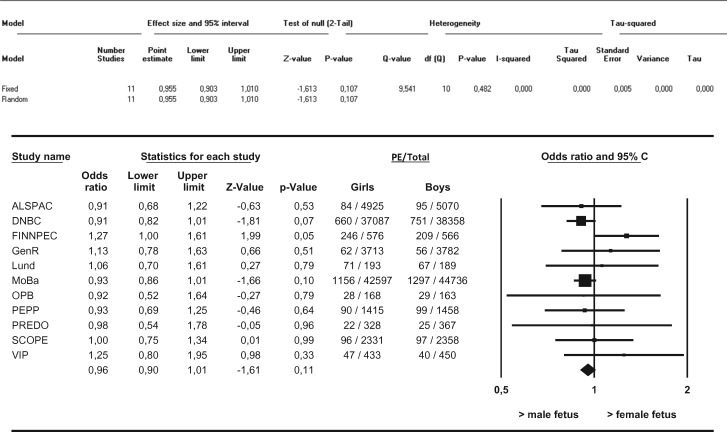
Associations between fetal sex and term *de novo* PE between female and male pregnancies Results from random-effects models. Data reflect Odds ratios (95% Confidence Interval) in which female term preeclampsia (PE) is compared to male term PE. Term PE was defined as gestational age ≥ 37+0 weeks at delivery.

**Figure 3.**
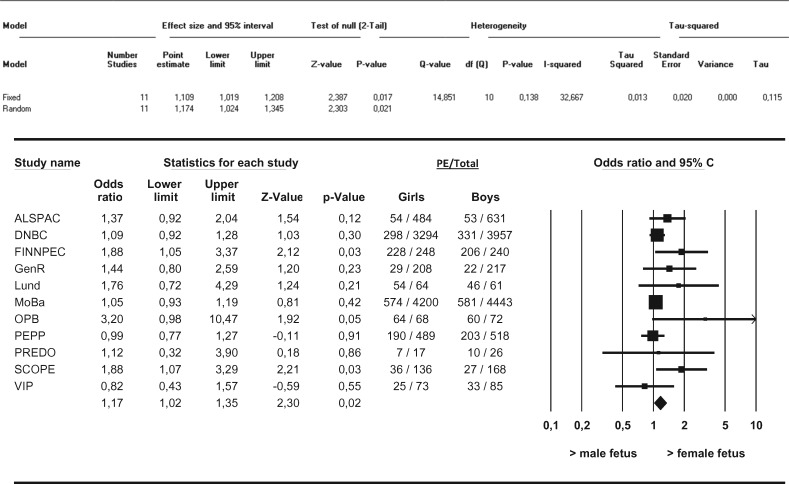
Associations between fetal sex and preterm *de novo* PE between female and male pregnancies Results from random-effects models. Data reflect Odds ratios (95% Confidence Interval) in which female preterm preeclampsia (PE) is compared to male preterm PE. Preterm PE was defined as gestational age < 37+0 weeks at delivery.

**Figure 4.**
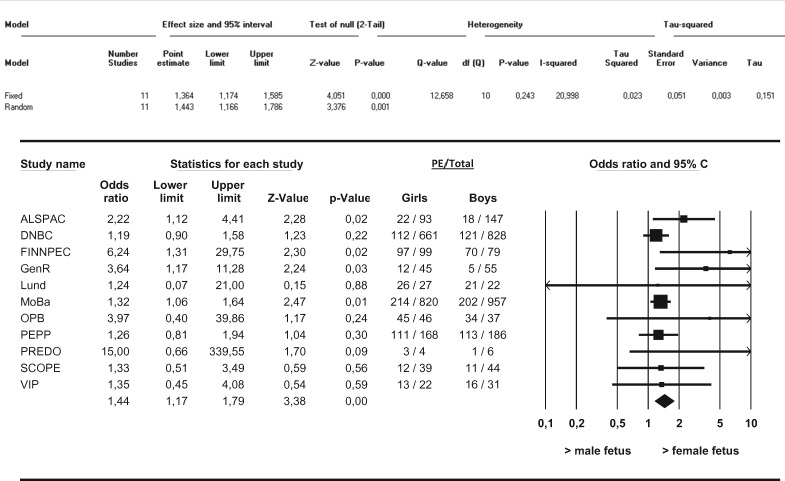
Associations between fetal sex and very preterm *de novo* PE between female and male pregnancies Results from random-effects models. Data reflect Odds ratios (95% Confidence Interval) in which female very preterm preeclampsia (PE) is compared to male very preterm PE. Very preterm PE was defined as gestational age < 34+0 weeks at delivery.

In this meta-analysis we observed no differences in the distribution of female versus male fetuses in the overall occurrence of PE (Figure 1). Furthermore, no differences in the distribution of female versus male fetuses with respect to *de novo* PE, superimposed PE or gestational hypertension were observed. We observed no differences in the female/male distribution with respect to term *de novo* PE (i.e. ≥ 37 weeks of gestation) (Figure 2). After stratification into preterm and very preterm *de novo* PE (i.e. < 37 weeks of gestation and < 34 weeks of gestation), differences in the distribution of female versus male fetuses in the occurrence of PE were observed. Female preterm PE was more prevalent than male preterm PE in pregnancies going beyond 22.0 weeks (OR 1.11, 95% CI 1.02–1.21, I^2 ^= 32.7%) (Figure 3). These results did not change after applying the leave-one-out method nor did restriction of these analyses to nulliparous women change the results. Very preterm PE was even more prevalent among pregnancies with a female fetus as compared with pregnancies with a male fetus (OR 1.36, 95% CI 1.17–1.59, I^2 ^= 21.0%) (Figure 4). Applying the leave-one-out method did not change the results nor did restriction of these analyses to nulliparous women (Supplementary Figures 1 and 2, available as Supplementary data at *IJE* online). Finally, no differences in the female/male distribution with respect to *de novo* PE between 34 and 37 weeks of gestation were observed. This suggests that the effects with respect to preterm PE are mainly determined by effects in the distribution of female versus male fetuses in very preterm PE (Supplementary Figure 3, available as Supplementary data at *IJE* online).

## Comment

Results from this large-scale meta-analysis of individual participants’ data show sexual dimorphic differences in the rates of PE subgroups, with preterm and very preterm PE being more prevalent among pregnancies with a female fetus as compared with pregnancies with a male fetus, and with no differences with respect to term PE. No differences in female/male distribution are observed in the overall risk of PE.

### Comparison with earlier studies and interpretation of main findings

PE has a deleterious impact on maternal and fetal morbidity, mortality and future health. It is a heterogeneous disorder with a complex aetiology and pathogenesis. Progress in the understanding of the disorder would be assisted greatly if subtypes could be characterized.^20^ Despite increasing evidence that maternal physiological functions are influenced in a fetal sex-specific manner during pregnancy, in most studies that assess potential pathophysiological mechanisms of PE, fetal sex has not been taken into account.

Previously, a large Norwegian population-based data study suggested that the sex ratio in PE displays a pattern strongly dependent on length of gestation.^5^ They showed that female babies were more frequent in PE with preterm delivery, whereas PE with term delivery was dominated by male offspring. Interestingly, when only assessing normotensive pregnancies, opposite results were observed with a male predominance in preterm births.^5^ Our results on PE are in line with theirs, indicating that fetal sex influences gestational age at delivery in pre-eclamptic pregnancies. These results are further supported by a recent study by Broere-Brown *et al.*^21^ showing fetal sex-specific differences in maternal vascular adaptation to pregnancy. They observed sex-specific differences in Doppler measurements of the uterine artery and sex-specific differences in both systolic and diastolic blood pressure patterns throughout pregnancy. Interestingly, differential effects according to the presence or absence of the placental syndromes, encompassing PE, IUGR and preterm birth, were observed. In pregnancies complicated by the placental syndromes women pregnant with a female fetus showed a higher blood pressure compared with women with a male fetus at the beginning of pregnancy. In contrast, by the end of the second trimester a shift in the male blood pressure pattern and female blood pressure pattern was observed. This resulted in a higher blood pressure for women with a male fetus compared with women with a female fetus at the end of pregnancy.^21^

Gestational age has been suggested as an indicator of subsets of PE with a different pathophysiology and with different acute and long-range outcomes for both mother and baby. We hypothesize that perhaps we might be looking at a biological phenomenon in which the observed sex-specific differences reflect a functional placental difference and subsequent response by the mother between the sexes with differential PE phenotypes as a result.

So what underlies the sexual dimorphism in PE? According to the two-stage model, impaired placentation including dysfunctional remodelling of the utero-placental arteries has been considered as powerful predisposing step in the aetiology of PE. This has especially been suggested for the early-onset subtype of PE.^3^^,^^22^ The first decidua-associated remodelling step should be initiated around implantation. Exposures at this stage might influence the risk of PE. Previously, it was hypothesized by Vatten *et al.* that a sex-specific susceptibility to the process of embryonic implantation could partly explain sexual dimorphic differences in PE.^5^ The so-called ‘cross-over’ in the sex ratio of PE was interpreted as an indication for the existence of two separate pathogenetic entities. The first pathogenetic entity would be associated with IUGR. Unfortunately, we did not have information available on the occurrence of IUGR to test this. The other pathogenetic entity proposed was that late-onset disease originated from abnormal implantation. Male embryos would be more susceptible to suboptimal implantation or abnormal placental development.^23^ This might imply that those pregnancies with a male embryo that are susceptible to develop PE due to impaired placentation may already have miscarried in the first trimester. The male fetuses that survive the period of placentation will thereby represent a relatively healthy group of fetuses leading to a female-biased prevalence of PE. Orzsack *et al.*^24^ showed higher first-trimester male miscarriage rates.^24^ Furthermore, lower first-trimester human chorionic gonadotrophin hormone concentrations (hCG) have been described for pregnancies with a male fetus compared with pregnancies with a female fetus.^25^ Since progesterone levels are higher in male fetuses and exert an inhibitory effect on hCG production, this may result in a lower hCG production by the male placenta and thereby results in a differential endometrial receptivity.^26^ HCG is proposed to promote angiogenesis in the uterine vasculature and to block any immunological action by the mother on foreign invading placental cells.^27^ This might also be related to earlier reported observations on a positive correlation between hCG levels, hyperemesis gravidarum and early-onset PE and fetal sex. Hyperemesis gravidarum is associated with higher levels of hCG and with an increased risk of early-onset PE.^28–30^ The presence of a female fetus is associated with hyperemesis.

The second stage of the two-stage model is associated with an exaggerated endothelial activation and a generalized hyperinflammatory state.^3^^,^^31^^,^^32^ Episodes of placental hypoxia or reperfusion result in oxidative stress, subsequent apoptotic and necrotic disruption of syncytial architecture and release of various components from the intervillous space into the maternal circulation that stimulates the production of inflammatory cytokines.^3^^,^^33^^,^^34^ Broere-Brown *et al.*^21^ previously showed that the placental release of circulating angiogenic and fibrinolytic factors differs according to fetal sex.^35^ They observed higher S-Flt1, PAI-2 and PlGF blood concentrations in cases of female as compared with male placentas. In pregnancies complicated by PE, spontaneous preterm birth or IUGR, however, no fetal sex-specific differences were observed. From this they concluded that perhaps other mechanisms causing these complications dominated the fetal sex effect.^35^ Muralimanoharan *et a*l.^36^ also presented evidence of sexual dimorphism in placentas from male fetuses compared with placentas from female foetuses, with higher levels of inflammatory, hypoxia and apoptotic molecules in males. This was observed in placental tissue of term pre-eclamptic pregnancies and is consistent with Vatten *et al.*^5^ In addition, they reported that in an obesogenic environment, primary trophoblasts derived from placentas of female fetuses have higher sensitivity to inflammatory stress compared with placentas of males. Interestingly, Minghetti *et al.*^37^ when studying preterm births, showed other results with higher umbilical cord blood levels of the oxidative stress biomarker 8-*iso*-PGF_2_α in male fetuses compared with female fetuses, using a natural twinning model.^37^ Isoprostanes are free radical-catalyzed prostaglandin-like products and considered as reliable markers of oxidative stress. In line with this, Yeganegi *et al.*^38^ and Challis *et al.*^39^ also demonstrated greater pro-inflammatory responses with a male fetus versus higher anti-inflammatory responses in pregnancies with a female fetus. They suggested that the male fetus exists in a relatively more ‘pro-inflammatory environment’ than the female fetus. This could account for the increased loss by miscarriage and spontaneous preterm birth with male fetuses. However, these latter three studies focused on preterm births in non-pre-eclamptic pregnancies and thereby are not completely pertinent to the distinct and multi-step entity of PE. We hypothesize that differences between pregnancies with male and female fetuses in the first (placental) but also second (systemic maternal) stage predispose to dimorphic differences in PE. Perhaps as previously suggested by Haig,^40^ PE is a disorder of failed interaction between two genetically different organisms. As PE is associated with long-term maternal health and in view of increasing interest in microchimerism (i.e. the long-term presence within an individual of a low level of cells derived from a different individual), the observed sexual dimorphic differences in the occurrence of PE might not be pertinent to pregnancy alone but also might have important long-term cardiovascular health implications for the mother.^2^^,^^4^

### Strengths and limitations

We performed a large meta-analysis with individual data from 11 studies participating in the CoLab consortium. We did not rely on published data, which limits any potential publication bias. The large number of participants enabled us to assess small effects. We presented results from random-effects models which allow heterogeneity in the true effect estimates between different populations and take between-study variation into account. By applying the leave-one-out method, we were able to determine the influence of any particular cohort on overall results. In agreement with other studies, we used the dating of gestational age at delivery as a proxy for the onset of PE, and not the time of first diagnosis. In a small subset of women (*n* = 1716) however, we did have information available on actual gestational age at PE diagnosis. These data were highly correlated with gestational age at birth (*r* = 0.89, *P* < 0.001). We therefore think it is unlikely that non-differential misclassification affected our effect estimates greatly.

Finally, we chose to exclude stillbirths since some studies did only include live-born infants whereas in other studies the presence of stillbirths could have been under-sampled (due to participation bias or loss-to-follow-up bias). Some stillbirths might have occurred before PE has been recognized clinically, or fetal sex may not have been determined in some of the very early stillbirths. Vatten *et al.*^5^ showed an increased risk of perinatal death in pre-eclamptic pregnancies in case of male fetuses. We had information available on 660 stillbirths. Additional analyses, however, in this subgroup showed no differences in the female/male distribution.

### Conclusion

In conclusion we found that there are fetal sex-specific differences in the occurrence of PE with a female dominance among preterm, but not term, pregnancies complicated by PE. Our results highlight the importance of fetal sex when studying placenta-mediated-diseases.

## Supplementary Data

Supplementary data are available at *IJE* online.

## Supplementary Material

Supplementary Data
