# Supplementary material for: Fetal sex-specific differences in gestational age at delivery in pre-eclampsia: a meta-analysis
Source: Int J Epidemiol. 2016 Sep 6;46(2):632–42. doi: 10.1093/ije/dyw178 (PMC5837300; doi:10.1093/ije/dyw178)
Supplement: Supplementary Data [file dyw178_supp.zip › ije-2016-01-0070-File010.docx]

**Supplemental Figure 3.** Associations between fetal sex and *de novo* 34-37 weeks of gestation PE between female and male pregnancies


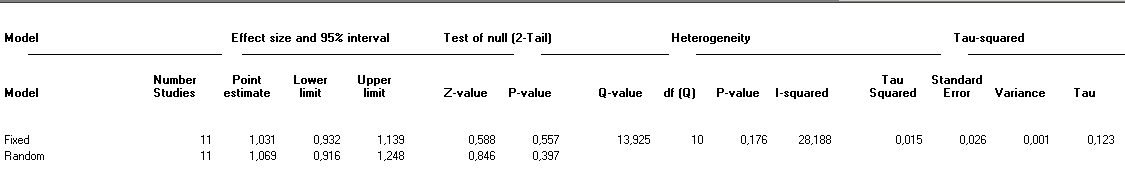

**PE/Total**

Results from random-effects models. Data reflect Odds ratios (95% Confidence Interval) in which female 34-37 weeks of gestation preeclampsia (PE) is compared to male 34-37 weeks of gestation PE.
