# Supplementary material for: Fetal sex-specific differences in gestational age at delivery in pre-eclampsia: a meta-analysis
Source: Int J Epidemiol. 2016 Sep 6;46(2):632–42. doi: 10.1093/ije/dyw178 (PMC5837300; doi:10.1093/ije/dyw178)
Supplement: Supplementary Data [file dyw178_supp.zip › ije-2016-01-0070-File008.docx]

**Supplemental Figure 1.** Associations between fetal sex and *de novo* preterm PE between female and male pregnancies: leave-one-out method

Data reflect Odds ratios (95% Confidence Interval) in which female preterm preeclampsia (PE) is compared to preterm male PE. Analyses were performed leaving out one cohort at a time. Preterm PE was defined as gestational age < 37+0 weeks at delivery.
